# Self-reported vaccination against SARS-CoV-2 and adverse events in multiple cohorts: Carolina Judith Klett-Tammen

**DOI:** 10.1093/eurpub/ckac131.405

**Published:** 2022-10-25

**Authors:** CJ Klett-Tammen, JK Heise, SM Soja, I Janzen, F Jenniches, Y Kemmling, G Behrens, TF Schulz, R Wegener, S Castell

**Affiliations:** 1 Epidemiology, Helmholtz Centre for Infection Research, Brunswick, Germany; 2 Study Centre, Helmholtz Centre for Infection Research, Hanover, Germany; 3 Department for Rheumatology and Immunology, Hanover Medical School, Hanover, Germany; 4 Institute of Virology, Hanover Medical School, Hanover, Germany; 5 Institute for Energy and Climate Research, IE, Forschungszentrum Jülich, Jülich, Germany; 6 TI Bioressources, Biodata und Digital Health, German Centre for Infection Research, Hanover, Germany

## Abstract

In two studies (“App-based infection assessment in RESIST (iAR)” and “Digital infection monitoring in persons living with immunodeficiency (DIMI)” ), we monitor health related items, as vaccination against SARS-CoV-2 and conduct syndromic surveillance of acute respiratory infections in high-risk populations, i.e. elderly persons and persons living with HIV, respectively. In a third very similar study (“Sensors for measuring aerosols and reactive gases to deduce health effects (SMARAGD)”) mainly healthy adults participate. To record incident or recurring transient health events, risk factors and further health data in real-time, we developed the eResearch system “PIA - Prospective Monitoring and Management App”. Recruitment for RESIST, SMARAGD and DIMI started in March 2021 and is ongoing. The questionnaire was presented in April 2022. Preliminary results include 86 participants from the three cohorts. In total, one indicated to be not vaccinated, none were vaccinated once, three (3.5%) twice, 63 (73.3%) three times and 19 (22.1%) four times. Participants reported the following adverse events after immunization (AEFI): after 40 applied doses with Vaxzevria® 24 AEFI (60%); after 158 doses of Comirnaty® 41 AEFI (26%); after 62 doses of Spikevax® 19 AEFI (30.7%); and after three doses of Janssen®, one AEFI (33.3%). In these cohorts, 20 (23.36%) participants stated having had a SARS-CoV-2 infection, of these 16 (80%) after the last vaccination dose, three (15%) before the first dose and one (5%) in between doses. Most participants were vaccinated three times, with Comirnaty being the most applied vaccine, as in officially reported numbers. AEFI varied according to vaccine and were higher than in the German surveillance system (1.64/1000 doses). Most infections were indicated to have been diagnosed after the booster vaccination. The results are limited by the small sample size and possible bias through self-reporting and social desirability regarding vaccination status.

**Key messages:**

• Overall, most participants were vaccinated with Comirnaty and had three doses of vaccine. Of the participants with a diagnosed SARS-CoV-2-infection, most got infected after the booster vaccine.

• The number of reported AEFI was higher than in the official surveillance in Germany.

